# Texture Analysis of Multi-Shot Echo-Planar Diffusion-Weighted Imaging in Head and Neck Squamous Cell Carcinoma: The Diagnostic Value for Nodal Metastasis

**DOI:** 10.3390/jcm8111767

**Published:** 2019-10-23

**Authors:** Jung Hyun Park, Yun Jung Bae, Byung Se Choi, Young Ho Jung, Woo-Jin Jeong, Hyojin Kim, Leonard Sunwoo, Cheolkyu Jung, Jae Hyoung Kim

**Affiliations:** 1 Department of Radiology, Ajou University School of Medicine, Ajou University Medical Center, Suwon 443-380, Korea; nadine16jhp@gmail.com; 2 Department of Radiology, Seoul National University Bundang Hospital, 82, Gumi-ro 173beon-gil, Bundang-gu, Seongnam 13620, Korea; leonard.sunwoo@gmail.com (L.S.); jck0097@gmail.com (C.J.); jaehkim@snu.ac.kr (J.H.K.); 3 Department of Otolaryngology-Head&Neck Surgery, Seoul National University Bundang Hospital, 82, Gumi-ro 173beon-gil, Bundang-gu, Seongnam 13620, Korea; entist@naver.com (Y.H.J.); safar@snubh.org (W.-J.J.); 4 Department of Pathology, Seoul National University Bundang Hospital, 82, Gumi-ro 173beon-gil, Bundang-gu, Seongnam 13620, Korea; hyojinkim7137@gmail.com

**Keywords:** squamous cell carcinoma, lymph node, texture analysis, diffusion-weighted imaging, apparent diffusion coefficient

## Abstract

Accurate assessment of nodal metastasis in head and neck squamous cell carcinoma (SCC) is important, and diffusion-weighted imaging (DWI) has emerged as a potential technique in differentiating benign from malignant lymph nodes (LNs). This study aims to evaluate the diagnostic performance of texture analysis using apparent diffusion coefficient (ADC) data of multi-shot echo-planar imaging-based DWI (msEPI-DWI) in predicting metastatic LNs of head and neck SCC. 36 patients with pathologically proven head and neck SCC were included in this study. A total of 204 MRI-detected LNs, including 176 subcentimeter-sized LNs, were assigned to metastatic or benign groups. Texture features of LNs were compared using independent *t*-test. Hierarchical cluster analysis was performed to exclude redundant features. Multivariate logistic regression and receiver operating characteristic analysis were performed to assess the diagnostic performance. The discriminative texture features for predicting metastatic LNs were complexity, energy and roundness. Areas under the curves (AUCs) for diagnosing metastasis in all/subcentimeter-sized LNs were 0.829/0.767 using complexity, 0.699/0.685 using energy and 0.671/0.638 using roundness, respectively. The combination of three features resulted in higher AUC values of 0.836/0.781. In conclusion, texture analysis of ADC data using msEPI-DWI could be a useful tool for nodal staging in head and neck SCC.

## 1. Introduction

Nodal metastasis in head and neck squamous cell carcinoma (SCC) is a major adverse prognostic factor requiring accurate assessment for optimal treatment [1,2,3]. However, nodal staging on imaging, such as computed tomography (CT) or conventional magnetic resonance imaging (MRI), has depended mainly on the size and morphologic criteria, which are limited in the detection of subcentimeter-sized nodal metastasis [2,4].

Diffusion-weighted imaging (DWI) has emerged as a potential MRI technique that can provide important biomarker in various tumors [5,6,7,8]. The quantifying degree of water diffusion by apparent diffusion coefficient (ADC) value is known to be correlated with the tumor cellularity, the proliferation index such as Ki-67, the presence of T-lymphocytes determined by CD-3 positive cell counts, the nucleic areas and even the human papilloma-virus status [5,6,7,8]. Using this attribute, many researchers have utilized the mean ADC measured in a single region-of-interest (ROI) located on the lymph nodes (LNs) for determining metastatic LNs [2,3,4,9,10]. The general consensus seems that the mean ADC in nodal metastasis from SCC is significantly lower than in benign LNs [2,11,12,13,14,15,16].

Nevertheless, there are still conflicting results being offered in several studies. Sumi et al. [8] reported that ADC in metastatic cervical LNs was significantly higher than in benign LNs, and Zhang et al. [17] verified this in an animal study. Thoeny et al. [18] assessed 4846 normal-sized pelvic LNs and concluded that ADC in malignant and benign LNs did not differ significantly; Lim et al. [19] derived the same result from analyzing cervical LNs. Moreover, most of the previous studies have included the nodal metastasis from nasopharyngeal SCC, which is known to have significantly lower ADC than SCC from other head and neck region. Thus, the ADC measurement in metastatic LNs including nasopharyngeal primary could have been biased toward lower ADC values than expected [20,21].

In addition, most of the previous studies have utilized single-shot echo-planar imaging-based DWI (ssEPI-DWI) in placing ROI on LNs. Since the head and neck have substantial susceptibility artifact leading to significant image distortion, the ADC measurement in ROI placed on ssEPI-DWI can be incorrect, especially for the subcentimeter-sized LNs. Recently, multi-shot EPI-based DWI (msEPI-DWI) has been introduced to provide non-distorted imaging by reducing susceptibility artifact, permitting more accurate ROI placement on the cervical LNs [22]. However, to our knowledge, there have been no studies utilizing msEPI-DWI in ADC assessment for cervical metastatic LNs.

In our study, we firstly adopted msEPI-DWI for the differentiation of the metastatic from benign cervical LNs using quantitative ADC measurement. Furthermore, we not only measured mean ADC in the LNs but performed texture analysis. Texture analysis is a technique that can effectively provide spatial information regarding variation of grey-level distribution and inter-relationships of voxels in the lesion [23], but never has been applied for the nodal staging of head and neck SCC. The purpose of our study was to determine the diagnostic performance of the whole-lesion volumetric texture analysis of ADC data derived from msEPI-DWI in predicting metastatic LNs of head and neck SCC.

## 2. Materials and Methods

### 2.1. Patients

**Figure 1:**
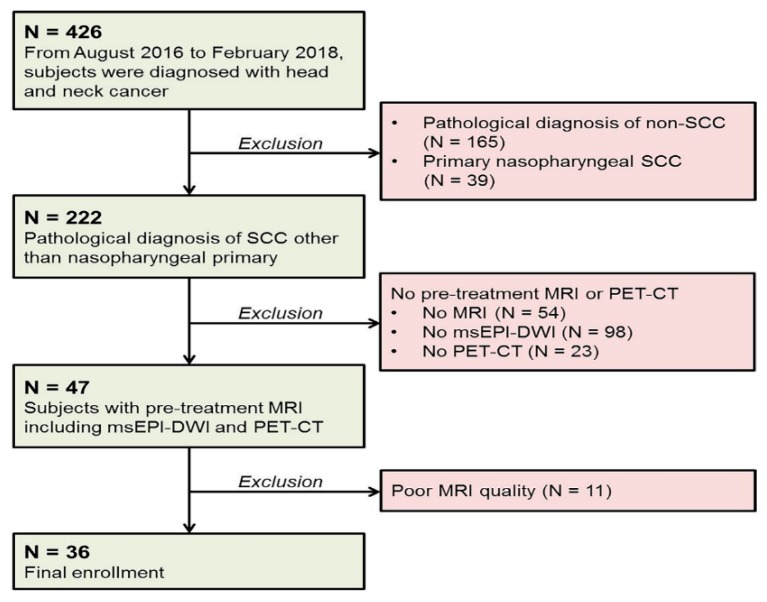
Patient flow diagram. Patients who were diagnosed with head and neck cancer and met the predetermined criteria were included in the final study population. Note—SCC, squamous cell carcinoma; MRI, magnetic resonance imaging; PET-CT, 2-deoxy-2-[fluorine-18]-fluoro-D-glucose (^18^F-FDG) positron emission tomography-computed tomography; msEPI-DWI, multi-shot echo-planar imaging-based diffusion-weighted imaging.

This retrospective study was approved by the Institutional Review Board of our hospital. Due to the retrospective nature of the study, the requirement for informed consent was waived. Between August 2016 and February 2018, a total of 426 patients were diagnosed with head and neck cancer in our institution. Among them, those who met the following criteria were included—(a) biopsy-proved primary SCC in the head and neck, (b) available pre-treatment MRI at 3T, including msEPI-DWI and (c) available pre-treatment 2-deoxy-2-[fluorine-18]-fluoro-D-glucose (^18^F-FDG) positron emission tomography (PET)-CT. The exclusion criteria were (a) pathological diagnosis of nasopharyngeal SCC or non-SCC, (b) unavailable pre-treatment MRI or ^18^F-FDG PET-CT and (c) inadequate MRI quality from artifact or subjects’ motion. The inclusion and exclusion processes are shown in Figure 1. Demographic data were collected via electronic medical record. As a result, of the 426 patients diagnosed with head and neck cancer during the study period, 36 patients (29 males and 7 females; age range, 28–79 years; mean age, 59 years) were finally included. All included patients were comprised of a single ethnicity of Asian.

### 2.2. MR Imaging Protocol

All MR imaging was acquired with a 3T instrument (Ingenia; Philips Healthcare, Best, The Netherlands) using a 32-channel sensitivity encoding head coil. The msEPI-DWI was performed in the axial plane using 2D navigated interleaved multi-shot EPI acquisition. The imaging parameters for DWI were—2 b-values of 0 and 1000 s/mm^2^; 3 orthogonal diffusion gradients; repetition time, 6400 ms; echo time, 65 ms; field-of-view, 220 × 220 mm^2^; acquisition matrix, 128 × 128; slice thickness, 3 mm; no slice gap; number of slice sections, 40; number of shots, 4; number of excitation, 2; scan time of 3 min 30 s. For morphologic evaluation, the following conventional sequences were obtained—axial turbo spin-echo T2-weighted image (T2-WI) with and without fat suppression, axial turbo spin-echo T1-WI, coronal turbo spin-echo T2-WI with fat suppression, followed by gadolinium-enhanced turbo spin-echo T1-WI in axial, coronal and sagittal planes.

### 2.3. Reference Standards

The nodal status was determined from the pre-treatment MRIs of all patients. Two board-certified neuro-radiologists (with 9 and 19 years of experience in head and neck imaging, respectively), who were blinded to the histological results, independently marked all visible LNs on the ADC map, considering anatomical information from other conventional sequences. Purely cystic LNs were excluded. Disagreements were resolved by consensus. All LNs were classified into metastatic or benign LNs, based on the histopathological results and/or ^18^F-FDG PET-CT results. Pathological–radiological correlation was made by subdividing the neck into six different levels according to the classification of the American Joint Committee on Cancer; this classification was used by the radiologists when reviewing the MR images, used by neck surgeons during the neck dissection and used by the pathologist when interpreting the specimen [24]. Conflicting cases were discussed at a weekly multidisciplinary tumor board including otorhinolaryngology-head and neck surgeons and pathologists, and consensus in nodal status was reached by matching the pre-operatively marked LNs on MRI with the biopsied and/or dissected specimens. In cases that the biopsy or surgery was not practical, the nodal status was evaluated on ^18^F-FDG PET-CT; PET-positive for metastasis was confirmed when LN showed higher FDG uptake than the background. For indeterminate LNs, follow-up imaging was used; LN showing interval change in the size after treatment and FDG uptake was designated as metastasis.

### 2.4. ^18^F-FDG PET-CT Protocol

^18^F-FDG PET-CT scans were acquired using a Discovery VCT scanner (GE Medical Systems, Milwaukee, WI, USA). All patients fasted for at least 6 hours prior to the scan and blood sugar levels were confirmed to be <120 mg/dL. 5.18 MBq/kg of ^18^F-FDG was administered intravenously in each patient 1 hour before PET-CT scanning. CT scans (3 mm slice thickness; tube voltage: 120 kVp; tube current-time product: 50–80 mAs depending on the patient’s weight) were performed to obtain anatomic information. PET scans were obtained from the skull base to the upper thigh level with a 128 × 128 matrix. Images were reconstructed with an ordered subsets expectation maximization iterative algorithm (2 iterations, 8 subsets). The standardized uptake values were calculated using lean body mass. 

### 2.5. Texture Analysis

**Figure 2:**
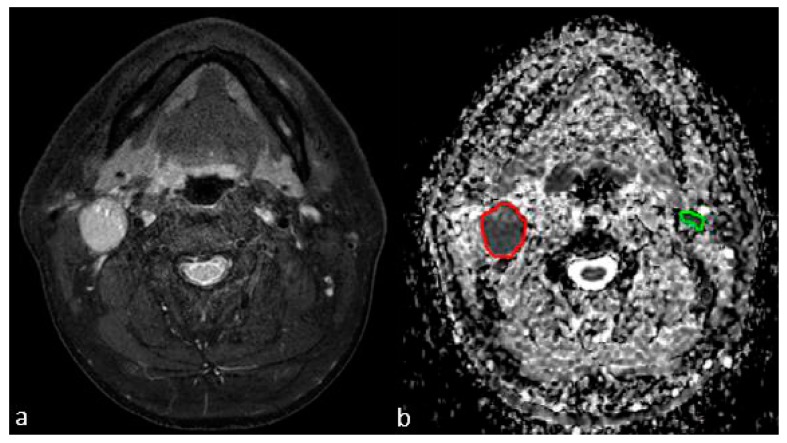
Images in a 54-year-old man with right tonsillar squamous cell carcinoma. (**a**) An enlarged lymph node (LN) larger than 1 cm is seen in right neck level IIA and another subcentimeter-sized LN is seen in left neck level IIA on the axial fat-suppressed T2-weighted image. (**b**) Region-of-interest is drawn over the enlarged right level II LN (red) and the left level II subcentimeter-sized LN (green) on the apparent diffusion coefficient (ADC) map, acquired in the axial plane using 2D navigated interleaved multi-shot echo-planar imaging-based diffusion-weighted imaging (msEPI-DWI).

Texture analysis was performed using Imaging Biomarker Explorer (IBEX) software [25]. ADC maps of all patients were exported to IBEX. To eliminate the partial volume averaging effect, LNs existing in three consecutive slices were considered for further processing. Polygonal ROIs were drawn over LNs on each axial ADC map under the consensus of two neuro-radiologists (Figure 2). Cystic/necrotic portion and fat hilum in the LNs were avoided while outlining ROI. Three-dimensional volume-of-interest (VOI) per LN was automatically created by the software.

For each VOI, 73 texture features from five categories were computed (Supplementary Materials, Table S1). First-order texture features, including shape/size (16 features) and intensity direct/histogram (19 features), were derived from histogram analysis. Second-order features were derived from gray-level co-occurrence matrix (22 features), gray level run length matrix (11 features) and neighborhood gray-tone difference matrix (5 features). Gray-level co-occurrence matric-based features were computed and analyzed separately using distance of 1 (d1), 4 (d4) and 7 (d7) pixels. Additional normalization accounting for the number of voxels was performed for the five volume-dependent features (busyness, coarseness, grey-level non-uniformity, run-length non-uniformity and energy) [26].

### 2.6. Statistical Analysis

All analyses were performed twice by including a) LNs of all sizes and b) subcentimeter-sized LNs with a maximal short-axis diameter of less than 1 cm. Continuous variables were expressed as the mean ± standard deviation. An independent *t*-test was used to compare the quantitative texture feature data between benign and metastatic LNs. Hierarchical cluster analysis was performed to identify the correlation between features; the features showing significant co-correlations with |Spearman correlation coefficient| >0.7 were considered as redundant and excluded from further analysis. Multivariate logistic regression with Cox proportional hazards model [27] was used to determine the significant independent features predicting metastatic LNs. Receiver operating characteristic (ROC) curves were generated for the significant independent features and the area under the curve (AUC) was calculated to assess the diagnostic performance. Lastly, the added value of the features was additionally determined by summing up the values of the significant texture features and creating an accordant ROC curve. The diagnostic performance of each ROC curve was compared using DeLong test [28]. A *p* value < 0.05 was considered statistically significant. Statistical analysis was performed using R v. 3.3.1. (R Foundation for Statistical Computing, Vienna, Austria) and SPSS v. 22.0 (SPSS, Chicago, IL, USA).

## 3. Results

### 3.1. Patients

Among the included patients, 17 patients had histopathologically confirmed oral cavity SCC, 17 patients had oropharyngeal SCC and 2 patients had hypopharyngeal SCC. Of the 36 patients, 28 patients underwent neck dissection, 5 patients underwent fine needle aspiration of the LNs under ultrasound guidance and 3 patients underwent ^18^F-FDG PET-CT only for nodal staging.

### 3.2. MRI Characteristics of LNs

A total of 204 benign or metastatic LNs were detected from the pre-treatment MRIs of 36 patients (level IA, *n* = 4; level IB, *n* = 34; level IIA, *n* = 121; level IIB, *n* = 24; level III, *n* = 16; level VA, *n* = 5). Among 204 LNs (neck dissection, *n* = 165; fine needle aspiration, *n* = 21; ^18^F-FDG PET/CT only, *n* = 18), 83 LNs were confirmed as metastasis (neck dissection, *n* = 60; fine needle aspiration, *n* = 13; ^18^F-FDG PET-CT only, *n* = 10); out of 176 subcentimeter-sized LNs (neck dissection, *n* = 155; fine needle aspiration, *n* = 16; ^18^F-FDG PET-CT only, *n* = 5), 58 LNs were confirmed as metastasis (neck dissection, *n* = 48; fine needle aspiration, *n* = 7; ^18^F-FDG PET-CT only, *n* = 3). The maximal short-axis diameter measured on T2-WI were 9.4 ± 0.6 mm (range = 6–39 mm) for metastatic LNs and 5.0 ± 0.1 mm for benign LNs (range = 3–27 mm) (*p* < 0.001). The VOI size for metastatic LNs was 993 ± 1430 voxels (range = 44–7329) and 217 ± 188 voxels (range = 42–1191) for benign LNs (*p* < 0.001). Regarding the subcentimeter-sized LNs only, the VOI size of metastatic LNs was 355 ± 304 voxels (range = 44–1757) and that of benign LNs was 211 ± 177 voxels (range = 42–1191) (*p* < 0.001). There were statistically significant differences in the maximal short-axis diameter and the VOI size between benign and metastatic LNs, both in all-sized LNs and subcentimeter-sized LNs; however, there was substantial overlap in values.

### 3.3. ADC Texture Analysis

The results of texture feature values according to benign and metastatic LNs are summarized in the Supplementary Materials (Tables S2 and S3). Total 103 out of the 157 ADC texture features in all-sized LNs and 94 out of the 157 features in subcentimeter-sized LNs showed significant differences between benign and metastatic LNs. Regarding the first-order ADC texture features, 66 out of 75 (in LNs of all sizes) and 65 out of 75 (in subcentimeter-sized LNs) features showed significant differences between benign and metastatic LNs. Out of the total 82 second-order ADC texture features, 37 (gray-level co-occurrence matrix, 30; gray level run length matrix, 6; neighborhood gray-tone difference matrix, 1) and 29 (gray-level co-occurrence matrix, 23; gray level run length matrix, 5; neighborhood gray-tone difference matrix, 1) features demonstrated significant differences in all- and subcentimeter-sized LNs, respectively. Many of the aforementioned features showed significant correlations with each other in the following cluster analysis. After excluding redundant features, 6 features (complexity, energy, global entropy, roundness, maximum probability (d7), short run low gray level emphasis) for all-sized LNs and 5 features (complexity, energy, global entropy, roundness, maximum probability (d7)) for subcentimeter-sized LNs were finally selected as the independent texture features for differentiation between benign and metastatic LNs.

### 3.4. Prediction of Metastatic LN by Texture Feature

Multivariate logistic regression using 6 and 5 independent features for all- and subcentimeter-sized LNs revealed that complexity, energy and roundness were the significant predictive factors for the metastatic LNs (Table 1).

**Figure 3:**
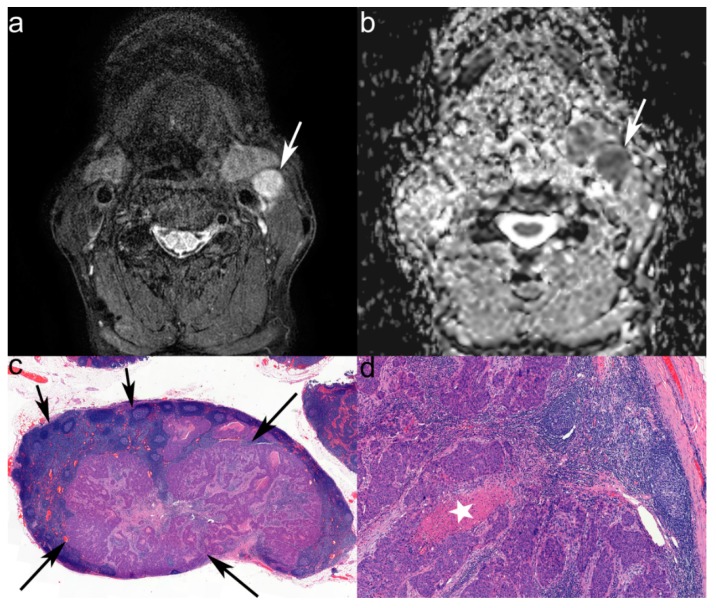
Images in a 62-year-old man with left tonsillar squamous cell carcinoma. (**a**) Axial T2-weighted image (T2-WI) with fat suppression shows about 1.8 cm sized lymph node (LN) in left neck level III (arrow). (**b**) This lymph node shows heterogeneously low apparent diffusion coefficient (ADC) value on the ADC map. The measured mean ADC value in this slice is 733 × 10^−6^ mm^2^/s. Under texture analysis, the measured complexity is 2022795, the normalized energy is 1363141 and the roundness was 0.303774, all of which are higher than the threshold for metastasis (245196, 667805 and 0.2473978, respectively). (**c**) On histopathologic examination, the metastatic deposit (long arrows) and the remaining normal lymphoid follicle structure (short arrows) can be seen (scan view, × 1). (**d**) On magnified view, the necrosis in the metastatic deposit can be demonstrated (asterisk, × 10).

Regarding LNs of all sizes, AUC for determining metastatic LNs was highest using complexity (0.829), followed by energy (0.699) and roundness (0.671). Regarding subcentimeter-sized LNs only, AUC was highest using complexity (0.767), followed by energy (0.685) and roundness (0.638). The estimated sensitivity and specificity at the optimal cut-off level for each value are summarized in Table 2. Figure 3 is a representative case of metastatic LN with correlative histopathology.

**Figure 4:**
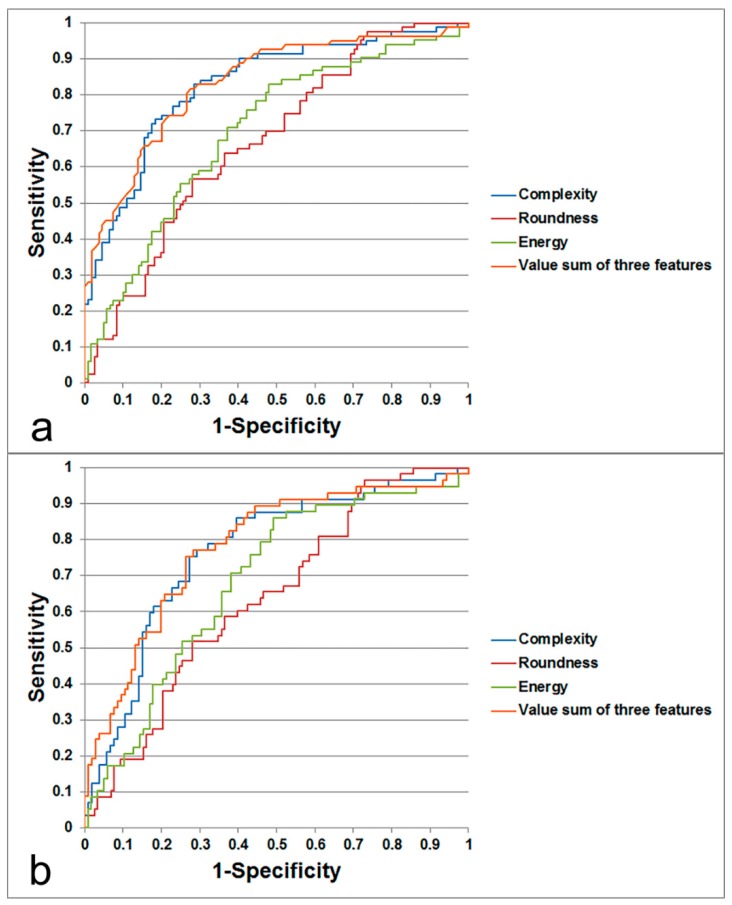
Receiver operating characteristic curves of texture features (complexity, energy and roundness) and the sum of all three features in all-sized lymph nodes (**a**) and subcentimeter-sized lymph nodes (**b**). The values of area under the curve (AUC) are presented in the Results.

When using a sum of the values from all three features (complexity, energy, roundness), instead of using complexity alone, a higher AUC value was obtained for both all-sized LNs (AUC using the sum of the three values, 0.836; AUC using complexity alone, 0.829) and subcentimeter-sized LNs (AUC using the sum of the three values, 0.781; AUC using complexity alone, 0.767); however, this was without statistical significance (*p* = 0.704 and 0.664) (Figure 4).

## 4. Discussion

In this study, we demonstrated that several features from the first- and second-order whole-lesion volumetric texture analysis of ADC data using msEPI-DWI were significantly different between metastatic and benign LNs in head and neck SCC. Among the features, even for subcentimeter-sized LNs, complexity, energy and roundness were significant predictive factors for nodal metastasis, with complexity being the single best predictive feature. The added value of combining these three features was shown in the present study; however, it failed to achieve statistical significance.

DWI has been incorporated in head and neck oncologic imaging to improve the discrimination of metastatic from benign LNs. The most recent meta-analysis study has concluded that the mean ADC values lower than the thresholds ranging from 749 to 1390 × 10^−6^ mm^2^/s could provide high diagnostic performance for metastatic LNs [11]. However, the reference studies in this meta-analysis have utilized ssEPI-DWI prone to the susceptibility artifact from the field inhomogeneity, producing significant image distortion in the head and neck [22]. Thus, ADC measurement based on ssEPI-DWI can be inaccurate, especially when the delineation of small nodal structure is challenging. To overcome this, the msEPI-DWI is increasingly used to reduce bandwidth-related EPI artifacts and phase errors to obtain homogeneous image without distortion [22]. The msEPI-DWI enables the accurate allocation of ROIs/ VOIs on the ADC map and the precise ADC measurement. However, the ADC values measured on msEPI-DWI is known to be different from those measured on ssEPI-DWI [22], which implies that the ADC threshold suggested by the previously published articles using ssEPI-DWI cannot be applied for the results from msEPI-DWI. Therefore, the research that can suggest specific ADC threshold using the msEPI-DWI is necessitated for the nodal staging in the head and neck.

Another point to discuss is that, there were studies that claimed the average ADC might not be lower in metastatic than benign LNs [8,17,18,19]. Inconsistent result from measuring mean nodal ADC can be supported by many reasons. First, nodal reactivity can decrease ADC to a level to that of metastatic LNs, because homogeneous lymphoid infiltration, organized germinal centers and fibrous stroma in reactive LNs can increase microstructural barriers [29]. Other benign abnormalities such as lipomatosis, sinus histocytosis and follicular hyperplasia can also impede diffusion, causing low ADC [18]. Second, nodal SCC can present morphological heterogeneity possessing dispersed metastatic deposits and micro-necrosis, which can contribute to the increase in the ADC value [29]. Moreover, in subcentimeter-sized metastatic LNs, small bunches of cancer cells may not create sufficient architectural change to affect mean ADC value [19]. In fact, there have been studies that showed higher mean ADC in tonsillar SCC than in normal tonsil, which seems contradictory to the results from nodal SCC when considering the similar histopathology of palatine tonsil and LNs [30,31]. Lastly, as we mentioned in the introduction, the inclusion of nodal metastasis from nasopharyngeal SCC could have been resulted in the biased assessment of ADC value. The primary nasopharyngeal SCC presents significant lower ADC than SCC from other head and neck regions [20], and the metastatic LNs from the nasopharyngeal SCC is known to present similar ADC values with primary SCC [21]. Therefore, we can speculate that the nodal metastasis from nasopharyngeal SCC might have significantly lower ADC that those from SCC from other head and neck region. Consequently, the overall inclusion of SCC from nasopharynx and from other parts of the head and neck for nodal ADC assessment could be misleading.

In our study, we firstly adopted msEPI-DWI and performed not only mean ADC measurement but whole-lesion volumetric texture analysis on the ADC map of the LNs in head and neck SCC other than nasopharyngeal primary. Texture analysis is a mathematical procedure to extract quantitative parameters from given images, which can detect subtle, sub-resolution changes in tumor morphology [32,33]. First-order texture features provide information related to the grey-level distribution without consideration of relative positions of the various grey levels within the image [23]. Second-order features, on the other hand, estimates the properties of two voxel values considering of the specific locations relative to each other [23]. In fact, the histogram analysis of MRI parameters from head and neck SCC has been proved more effective in identifying the histopathology of the tumor such as p53 and p16 expression, tumor proliferative index such as Ki-67, the cell counts and the nucleic areas, compared to the studies that only used averaged values of the parameters [34,35]. Considering the concrete spatial data that both the first- and second-order texture analyses can offer, we believed that our study could provide a comprehensive analysis of nodal ADC values, expanding knowledge from histogram analysis, to discriminate metastatic from benign LNs.

As a result, we found three texture features with the potential to be the independent significant factors for predicting metastatic LNs in head and neck SCC with high diagnostic performance. First, we found that complexity was the best discriminative factor for both large- and subcentimeter-sized LNs, which is a second-order feature related to non-uniform and rapid changes in grey levels [36]. A texture with rapid spatial changes in signal intensity is more likely to be complex; hence, complexity is a feature that quantifies spatial heterogeneity [37,38]. We postulated that metastatic LNs would have higher intranodal heterogeneity in ADC values due to the presence of subpopulation of dedifferentiated cells with higher cellularity in a mixture with necrosis and normal cells [29,39]. Our results showed that metastatic LNs had a higher value of complexity, which supports this assumption. Second, roundness was significantly elevated in large- and subcentimeter-sized metastatic LNs. Metastatic LNs are known to be rounder and less rentiform compared to benign LNs. Indeed, Schacht et al. [40] showed that circularity texture feature was higher in metastatic axillary LNs in breast cancer, using dynamic contrast-enhanced MRI-based texture analysis. Similar result was achieved in another study showing increased roundness in metastatic mediastinal LN in lung cancer patients [41]. Therefore, our result is concordant with previous reports. Third, we found that, regardless of nodal size, higher value of energy—a first-order texture feature—could differentiate metastatic from benign LNs. The energy feature only measures the uniformity of the intensity level distribution. If the value is high, then there may be a distribution of a small number of intensity levels [42]. We presumed that tumor cell nests coming into the nodal sinus in flocks could increase the energy by increasing the number of same intensity levels in a certain VOI and simultaneously increase the complexity by increasing the spatial changes in intensities of the neighboring voxels. It is worth noting that there was a contradictory result reporting decreased energy value in metastatic axillary LNs; however, this result was obtained from dynamic contrast-enhanced MRI-based texture analysis [40]. Further study is needed to better understand the different texture feature results in different MRI sequences.

Compared with our study, the previous studies using PET-MRI showed higher sensitivities (92–100%) and specificities (77–93%) for detecting metastatic LNs [43]. However, the sensitivity and specificity were dropped to 66% and 87%, respectively, for small LNs with clinically negative neck examination [43], which is lower than our diagnostic performance regarding subcentimeter-sized LNs. Therefore, we might assume that the texture analysis of ADC data could be superior to PET-MRI in assessing subcentimeter-sized LNs. In addition, previous studies using ADC data from DWI without texture analysis reported mixed results of sensitivities (54.5–97.1%) and specificities (65.3–96.3%) [11]. We presume that this is likely due to the different number of study population and the differences in the image protocol for DWI. Thus, for proper assessment, the validation of the optimal protocol for DWI should be performed.

Our study has several limitations. First, this was a retrospective study with inclusion of 36 patients with head and neck SCC. This was because a larger number of patients were excluded due to the lack of msEPI-DWI and primary nasopharyngeal SCC [20]. However, by including all measurable LNs, we could analyze a substantial number of benign and metastatic LNs. Second, this was a single-center study using a certain type of MR scanner with identical protocol. Multi-center study with different MR scanners and protocols is warranted before it is introduced into routine clinical practice. Third, we did not utilize conventional MRI such as T1- or T2-WI in the texture in combination with ADC data. Texture features derived from T2-WI could be a useful imaging biomarker for nodal characterization, predicting the treatment response after chemo-radiation [44], which might suggest that the features from conventional MRI may add the diagnostic value to the DWI. Therefore, further study using multi-parametric MRI features would be beneficial. Fourth, ^18^F-FDG PET-CT was considered as the reference standard of nodal metastasis for 3 out of 36 patients. Of these 3 patients, total 18 LNs were included in the study and 10 LNs (standardized uptake value range, 4.5–11.2) were considered as metastasis. However, we think that false positive/negative findings of ^18^F-FDG PET-CT might have had little influence on our study result, since most of the included LNs showed either no hyper-metabolism or very high standardized uptake value with strong radiologic evidence of metastasis. Fifth, the values of complexity, energy and roundness in benign and metastatic LN groups showed some overlap. Further study with a larger population and various kinds of head and neck SCC might be needed before their use in daily practice. Lastly, the diagnostic performance based on AUC, sensitivity and specificity was still lower in subcentimeter-sized LNs than in LNs of all sizes. We speculate that the difference in texture features reflecting nodal heterogeneity could be less prominent in subcentimeter-sized LNs.

In conclusion, we identified ADC texture features from msEPI-DWI that were significantly different between metastatic and benign LNs the in head and neck SCC. Complexity, energy and roundness were three independent significant discriminative texture features for predicting metastatic LNs, even in subcentimeter-sized LNs, with high diagnostic performance. Therefore, texture analysis of ADC data using msEPI-DWI could be a useful tool for nodal staging in head and neck SCC.

## Figures and Tables

**Table 1:** Results of multivariate logistic regression analysis with Cox proportional hazards model using independent texture features.

Group	Texture Feature	Coefficient	*p* Value	OR	95% CI of OR
**All-sized LNs**	Complexity	0.22 (×10^−5^)	<0.001	1.000002	1.000001–1.000004
	Energy	0.19 (×10^−5^)	0.014	1.000002	1.0000004–1.000004
	Roundness	4.64	0.008	103.56	3.35–3675.25
**Subcentimeter-sized LNs**	Complexity	0.20 (×10^−5^)	0.001	1.0000020	1.0000008–1.000004
	Energy	0.23 (×10^−5^)	0.001	1.000002	1.0000009–1.000004
	Roundness	4.76	0.008	116.88	3.39–4675.74

Note.—CI, confidence interval; OR, odds ratio; LN, lymph node.

**Table 2:** Cutoff value and corresponding sensitivity, specificity for ADC texture features.

Diagnostic Performance	All-Sized LNs			Subcentimeter-Sized LNs		
	Complexity	Energy	Roundness	Complexity	Energy	Roundness
**AUC**	0.829	0.699	0.671	0.767	0.685	0.638
**Ben mean (SD)**	224278.15 (291381.92)	675113.33 (239553.38)	0.24 (0.11)	213192.64 (266248.09)	679498.11 (240590.86)	0.24 (0.11)
**Met mean (SD)**	1522108.84 (3090971.29)	850806.46 (303868.16)	0.30 (0.10)	506720.89 (449132.79)	825260.67 (288103.42)	0.28 (0.10)
**Cutoff value**	245196	667805	0.2473978	211147	677054.9	0.2473978
**Sensitivity (%)**	82.93	72.29	65.06	78.95	70.69	60.34
**Specificity (%)**	71.56	60.33	60.33	67.92	61.86	60.17

Note—ADC, apparent diffusion coefficient; LN, lymph node; AUC, area under the receiver-operating characteristic curve; Ben, benign LNs; Met, metastatic LNs; SD, standard deviation. Sensitivity and Specificity were obtained at the optimal cut-off values.

## Supplementary Materials

The following are available online at https://www.mdpi.com/2077-0383/8/11/1767/s1, Table S1: Texture features analyzed in this study and their abbreviations, Table S2: Comparison of ADC texture features between benign and metastatic LNs of all sizes., Table S3: Comparison of ADC texture features between benign and metastatic subcentimeter-sized LNs.
