# Supplementary material for: Texture Analysis of Multi-Shot Echo-Planar Diffusion-Weighted Imaging in Head and Neck Squamous Cell Carcinoma: The Diagnostic Value for Nodal Metastasis
Source: J Clin Med. 2019 Oct 23;8(11):1767. doi: 10.3390/jcm8111767 (PMC6912832; doi:10.3390/jcm8111767)
Supplement: Supplementary file 1 [file jcm-08-01767-s001.pdf]

Table S1. Texture features analyzed in this study and their abbreviations.

Feature category	Feature (abbreviation)
**Shape/Size**	Compactness 1
	Compactness 2
	Convex
	Convex Hull Volume
	Convex Hull Volume 3D
	Mass
	Maximum 3D Diameter (Max3DDiameter)
	Mean Breadth
	Number Of Voxels (NOV)
	Orientation
	Roundness
	Spherical Disproportion (Spherical Disp)
	Sphericity
	Surface Area
	Surface Area Density
	Volume
**Intensity**	Kurtosis
**direct/histogram**	Skewness
	Range
	Variance
	Root Mean Square
	Energy
	Global Entropy
	Global Maximum (Global Max)
	Global Mean
	Global Median
	Global Minimum (Global Min)
	Global Standard Deviation (Global SD)
	Global Uniformity
	Mean Absolute Deviation
	Median Absolute Deviation
	Percentile
	Percentile Area
	Quantile
	Inter-QuartileRange
**GLCM**	Auto-Correlation
	Cluster Prominence
	Cluster Shade
	Cluster Tendency

	Contrast
	Correlation
	Difference Entropy
	Dissimilarity
	Energy
	Entropy
	Homogeneity
	Homogeneity 2
	Information Measure Correlation 1 (InfoMeasureCorr 1)
	Information Measure Correlation 2 (InfoMeasureCorr 2)
	Inverse Difference Moment Norm (InvDifMN)
	Inverse Difference Norm (InvDifN)
	Inverse Variance
	Maximum Probability (Max Probability)
	Sum Average
	Sum Entropy
	Sum Variance
	Variance
**GLRLM**	Gray Level Non-uniformity (GLN)
	High Gray Level Run Emphasis (HGRE)
	Long Run Emphasis (LRE)
	Long Run High Gray Level Emphasis (LRHGE)
	Long Run Low Gray Level Emphasis (LRLGE)
	Low Gray Level Run Emphasis (LGRE)
	Run Length Non-uniformity (RLN)
	Run Percentage (RP)
	Short Run Emphasis (SRE)
	Short Run High Gray Level Emphasis (SRHGE)
	Short Run Low Gray Level Emphasis (SRLGE)
**NDM**	Busyness
	Coarseness
	Complexity
	Contrast 1
	Texture Strength

Note. — GLCM, gray-level co-occurrence matrix; GLRLM, gray-level run-length matrix; NDM, neighborhood gray-tone difference matrix

Table S2. Comparison of ADC texture features between benign and metastatic LNs of all sizes.

Feature	Benign LNs (n = 121)	Metastatic LNs (n = 83)	*P* value
**Compactness 1**	0.10 (0.03)	0.21 (0.16)	<.001*
**Compactness 2**	0.41 (0.14)	0.46 (0.15)	.03*
**Convex**	0.93 (0.04)	0.94 (0.04)	.124
**Convex Hull Volume**	0.50 (0.45)	2.35 (3.53)	<.001*
**Convex Hull Volume 3D**	0.49 (0.59)	2.63 (4.47)	<.001*
**Mass**	0.42 (0.31)	2.07 (3.07)	<.001*
**Max3DDiameter**	1.44 (0.57)	2.07 (0.97)	<.001*
**Mean Breadth**	1.16 (0.43)	1.71 (0.88)	<.001*
**NOV**	216.76 (188.36)	993.37 (1430.64)	<.001*
**Orientation**	-4.88 (42.54)	5.20 (37.07)	.082
**Roundness**	0.24 (0.11)	0.30 (0.10)	<.001*
**Spherical Disp**	1.38 (0.16)	1.34 (0.19)	.122
**Sphericity**	0.74 (0.08)	0.76 (0.09)	.052
**Surface Area**	3.79 (2.32)	9.22 (9.35)	<.001*
**Surface Area Density**	7.39 (1.03)	5.64 (1.74)	<.001*
**Volume**	0.45 (0.39)	2.15 (3.28)	<.001*
**Energy**	675113.33 (239553.38)	850806.46 (303868.16)	<.001*
**Global Entropy**	5.42 (0.31)	5.64 (0.34)	<.001*
**Global Max**	1778.80 (431.29)	2075.65 (502.47)	<.001*
**Global Mean**	770.32 (137.47)	868.63 (167.58)	<.001*
**Global Median**	754.80 (134.82)	847.54 (169.68)	<.001*
**Global Min**	101.58 (144.80)	158.81 (191.82)	.016*
**Global SD**	245.30 (56.44)	254.29 (64.30)	.293
**Global Uniformity ($\times 10^{-3}$)**	29.50 (6.54)	25.64 (6.74)	<.001*
**Inter-Quartile Range**	263.59 (77.77)	297.91 (97.98)	.006*
**Kurtosis**	6.21 (6.79)	5.64 (2.81)	.469
**Mean Absolute Deviation**	178.84 (44.47)	191.32 (52.33)	.069
**Median Absolute Deviation**	128.78 (38.87)	146.51 (47.52)	.004*
**5th Percentile**	401.45 (171.32)	500.38 (195.54)	<.001*
**10th Percentile**	498.87 (146.76)	580.92 (181.40)	<.001*
**15th Percentile**	556.06 (134.81)	633.50 (178.17)	.001*
**20th Percentile**	595.96 (134.15)	673.62 (174.87)	<.001*
**25th Percentile**	629.50 (130.30)	706.55 (171.28)	<.001*
**30th Percentile**	658.69 (129.66)	735.71 (169.88)	<.001*
**35th Percentile**	683.92 (130.17)	765.15 (169.33)	<.001*
**40th Percentile**	708.28 (131.16)	791.92 (167.81)	<.001*
**45th Percentile**	731.66 (133.08)	818.75 (167.59)	<.001*
**50th Percentile**	754.80 (134.82)	847.54 (169.68)	<.001*
**55th Percentile**	778.48 (136.10)	874.37 (171.40)	<.001*
**60th Percentile**	802.51 (139.59)	902.61 (172.63)	<.001*
**65th Percentile**	830.09 (142.77)	933.53 (174.54)	<.001*

**70th Percentile**	859.31 (146.22)	967.98 (178.26)	<.001*
**75th Percentile**	893.09 (152.94)	1004.46 (181.36)	<.001*
**80th Percentile**	933.34 (159.15)	1048.74 (185.55)	<.001*
**85th Percentile**	986.30 (169.51)	1104.43 (188.28)	<.001*
**90th Percentile**	1058.53 (180.78)	1179.88 (192.42)	<.001*
**95th Percentile**	1185.76 (200.18)	1312.65 (209.69)	<.001*
**0.025 Quantile**	303.87 (184.57)	423.43 (207.46)	<.001*
**0.25 Quantile**	629.50 (130.30)	706.55 (171.28)	<.001*
**0.5 Quantile**	754.80 (134.82)	847.54 (169.68)	<.001*
**0.75 Quantile**	893.09 (152.94)	1004.46 (181.36)	<.001*
**0.975 Quantile**	1321.91 (223.54)	1446.44 (224.89)	<.001*
**Range**	1677.22 (431.06)	1916.84 (568.64)	.001*
**Root Mean Square**	810.23 (137.11)	908.16 (162.40)	<.001*
**Skewness**	0.61 (0.92)	0.77 (0.62)	.165
**Variance**	63331.70 (30689.10)	68747.63 (35545.04)	.247
**5th Percentile Area**	400.28 (158.61)	495.91 (188.20)	<.001*
**10th Percentile Area**	488.61 (146.97)	578.34 (171.62)	<.001*
**15th Percentile Area**	546.68 (135.85)	632.50 (164.73)	<.001*
**20th Percentile Area**	586.50 (133.75)	665.32 (174.25)	<.001*
**25th Percentile Area**	620.70 (130.60)	697.53 (171.75)	<.001*
**30th Percentile Area**	649.70 (129.93)	727.17 (170.18)	<.001*
**35th Percentile Area**	675.20 (130.80)	756.36 (168.77)	<.001*
**40th Percentile Area**	699.59 (131.10)	783.17 (167.47)	<.001*
**45th Percentile Area**	722.58 (132.38)	810.62 (167.98)	<.001*
**50th Percentile Area**	746.14 (134.98)	838.58 (168.86)	<.001*
**55th Percentile Area**	770.30 (136.16)	866.12 (171.09)	<.001*
**60th Percentile Area**	794.04 (139.50)	894.36 (172.73)	<.001*
**65th Percentile Area**	821.72 (142.74)	925.05 (174.37)	<.001*
**70th Percentile Area**	850.96 (147.05)	958.59 (177.46)	<.001*
**75th Percentile Area**	884.94 (152.89)	995.73 (181.40)	<.001*
**80th Percentile Area**	924.84 (159.07)	1039.83 (185.87)	<.001*
**85th Percentile Area**	977.15 (168.82)	1095.63 (188.69)	<.001*
**90th Percentile Area**	1047.95 (180.87)	1170.64 (192.37)	<.001*
**95th Percentile Area**	1171.84 (197.66)	1300.69 (208.81)	<.001*
**Auto-Correlation (d1)**	1440.57 (507.66)	1795.07 (667.17)	<.001*
**Auto-Correlation (d4)**	1438.30 (527.49)	1806.98 (670.65)	<.001*
**Auto-Correlation (d7)**	1431.31 (607.75)	1814.81 (708.52)	<.001*
**Cluster Prominence (d1)**	502843.69 (533650.77)	592506.04 (570655.53)	.253
**Cluster Prominence (d4)**	349229.23 (442311.84)	380630.76 (382452.54)	.6
**Cluster Prominence (d7)**	356888.36 (480589.35)	381940.28 (338085.85)	.682
**Cluster Shade (d1)**	2097.16 (4511.93)	2996.57 (4232.39)	.153
**Cluster Shade (d4)**	1755.86 (3952.12)	2116.32 (2848.19)	.476
**Cluster Shade (d7)**	2092.85 (4290.07)	2249.82 (2966.53)	.773

**Cluster Tendency (d1)**	299.23 (163.77)	341.76 (195.20)	.094
**Cluster Tendency (d4)**	266.55 (136.99)	290.94 (157.49)	.241
**Cluster Tendency (d7)**	289.44 (167.38)	293.09 (151.22)	.874
**Contrast (d1)**	134.25 (62.33)	146.41 (79.11)	.222
**Contrast (d4)**	260.07 (131.59)	249.45 (124.58)	.564
**Contrast (d7)**	422.72 (334.17)	309.00 (183.92)	.005*
**Correlation (d1)**	0.37 (0.14)	0.40 (0.10)	.119
**Correlation (d4)**	0.06 (0.14)	0.10 (0.12)	.108
**Correlation (d7)**	0.01 (0.25)	0.02 (0.18)	.879
**Difference Entropy (d1)**	4.44 (0.31)	4.50 (0.38)	.227
**Difference Entropy (d4)**	4.78 (0.30)	4.86 (0.36)	.085
**Difference Entropy (d7)**	4.23 (0.87)	4.75 (0.56)	<.001*
**Dissimilarity (d1)**	8.23 (1.94)	8.57 (2.41)	.279
**Dissimilarity (d4)**	12.05 (3.20)	11.82 (3.17)	.606
**Dissimilarity (d7)**	15.38 (6.88)	13.18 (4.38)	.011*
**Energy (d1) (×10^{-3})**	2.99 (1.31)	2.18 (1.23)	<.001*
**Energy (d4) (×10^{-3})**	6.67 (4.98)	3.40 (3.82)	<.001*
**Energy (d7)**	0.05 (0.10)	0.01 (0.03)	.007*
**Entropy (d1)**	8.95 (0.59)	9.58 (0.72)	<.001*
**Entropy (d4)**	7.69 (1.01)	8.92 (1.16)	<.001*
**Entropy (d7)**	5.70 (1.80)	7.81 (1.92)	<.001*
**Homogeneity (d1)**	0.23 (0.04)	0.22 (0.04)	.759
**Homogeneity (d4) (×10^{-3})**	167.40 (34.45)	169.40 (34.75)	.693
**Homogeneity (d7)**	0.14 (0.05)	0.15 (0.04)	.11
**Homogeneity 2 (d1)**	0.14 (0.03)	0.14 (0.04)	.89
**Homogeneity 2 (d4) (×10^{-3})**	90.69 (28.62)	92.30 (28.19)	.685
**Homogeneity 2 (d7)**	0.07 (0.04)	0.08 (0.03)	.152
**InfoMeasureCorr 1 (d1)**	-0.18 (0.06)	-0.14 (0.07)	<.001*
**InfoMeasureCorr 1 (d4)**	-0.42 (0.15)	-0.26 (0.18)	<.001*
**InfoMeasureCorr 1 (d7)**	-0.68 (0.19)	-0.43 (0.27)	<.001*
**InfoMeasureCorr 2 (d1)**	0.90 (0.06)	0.83 (0.12)	<.001*
**InfoMeasureCorr 2 (d4)**	0.98 (0.05)	0.86 (0.19)	<.001*
**InfoMeasureCorr 2 (d7)**	0.99 (0.09)	0.92 (0.15)	<.001*
**InvDifMN (d1) (×10^{-3})**	987.54 (5.45)	986.45 (6.95)	.211
**InvDifMN (d4) (×10^{-3})**	976.48 (11.03)	977.37 (10.62)	.565
**InvDifMN (d7)**	0.95 (0.09)	0.96 (0.11)	.699
**InvDifN (d1)**	0.93 (0.01)	0.93 (0.02)	.304
**InvDifN (d4) (×10^{-3})**	899.70 (22.92)	901.30 (22.91)	.624
**InvDifN (d7)**	0.87 (0.09)	0.88 (0.10)	.442
**Inverse Variance (d1)**	0.14 (0.03)	0.14 (0.04)	.588
**Inverse Variance (d4)**	0.09 (0.03)	0.10 (0.03)	.856
**Inverse Variance (d7)**	0.08 (0.05)	0.09 (0.04)	.331
**Max Probability (d1) (×10^{-3})**	11.53 (5.57)	9.53 (8.62)	.046*

**Max Probability (d4)**	0.02 (0.01)	0.01 (0.01)	<.001*
**Max Probability (d7)**	0.05 (0.10)	0.02 (0.03)	.002*
**Sum Average (d1)**	73.74 (12.69)	82.07 (15.90)	<.001*
**Sum Average (d4)**	74.67 (13.11)	83.30 (15.84)	<.001*
**Sum Average (d7)**	74.80 (16.30)	83.28 (18.56)	.001*
**Sum Entropy (d1)**	5.88 (0.36)	6.02 (0.39)	.009*
**Sum Entropy (d4)**	5.59 (0.38)	5.82 (0.40)	<.001*
**Sum Entropy (d7)**	4.69 (1.09)	5.48 (0.79)	<.001*
**Sum Variance (d1)**	5061.26 (1886.68)	6372.34 (2480.55)	<.001*
**Sum Variance (d4)**	5208.49 (1997.06)	6540.26 (2505.15)	<.001*
**Sum Variance (d7)**	5512.16 (2288.68)	6763.75 (2596.12)	<.001*
**Variance (d1)**	299.23 (163.77)	341.76 (195.20)	.094
**Variance (d4)**	266.55 (136.99)	290.94 (157.49)	.241
**Variance (d7)**	289.44 (167.38)	293.09 (151.22)	.874
**Busyness (×10^{-3})**	0.97 (3.59)	0.12 (0.19)	.215
**Coarseness (×10^{-5})**	6.25 (7.99)	9.47 (6.90)	.63
**Complexity**	224278.15 (291381.92)	1522108.84 (3090971.29)	<.001*
**Contrast 1**	19666.24 (39131.58)	10914.41 (26586.01)	.083
**Texture Strength**	435.63 (388.67)	478.67 (411.12)	.461
**GLN (×10^{-3})**	0.24 (0.19)	0.10 (0.09)	<.001*
**HGRE**	1595.27 (546.17)	1964.72 (685.88)	<.001*
**LRE**	1.24 (0.11)	1.27 (0.12)	.082
**LRHGE**	1910.77 (600.64)	2408.97 (849.93)	<.001*
**LRLGE**	0.02 (0.03)	0.02 (0.06)	.927
**LGRE (×10^{-3})**	9.41 (11.48)	6.46 (12.43)	.082
**RLN (×10^{-3})**	5.80 (3.64)	2.72 (2.38)	<.001*
**RP**	0.93 (0.02)	0.93 (0.03)	.093
**SRE (×10^{-3})**	950.76 (17.09)	947.01 (17.76)	.131
**SRHGE**	1529.25 (536.56)	1875.42 (661.04)	<.001*
**SRLGE (×10^{-3})**	8.12 (9.11)	5.04 (8.09)	.014*

Note. — ADC, apparent diffusion coefficient; LN, lymph node; Other abbreviations, summarized in Table 1. a. All values are expressed as mean (standard deviation). b. The significance threshold for difference was set at a *P* value less than .05, according to independent *t*-test. * means statistically significant.

Table S3. Comparison of ADC texture features between benign and metastatic subcentimeter-sized LNs.

Feature	Benign LNs (n = 118)	Metastatic LNs (n = 58)	*P* value
**Compactness 1**	0.09 (0.03)	0.13 (0.06)	<.001*
**Compactness 2**	0.41 (0.14)	0.46 (0.15)	.051
**Convex**	0.93 (0.04)	0.94 (0.03)	.032*
**Convex Hull Volume**	0.49 (0.42)	0.83 (0.75)	<.001*
**Convex Hull Volume 3D**	0.48 (0.57)	0.82 (0.87)	.002*
**Mass**	0.41 (0.29)	0.77 (0.67)	<.001*
**Max3DDiameter**	1.44 (0.56)	1.67 (0.57)	.012*
**Mean Breadth**	1.16 (0.42)	1.34 (0.42)	.009*
**NOV**	211.28 (176.59)	355.52 (303.74)	<.001*
**Orientation**	-6.02 (42.17)	2.70 (39.08)	.188
**Roundness**	0.24 (0.11)	0.28 (0.10)	.005*
**Spherical Disp**	1.38 (0.16)	1.33 (0.16)	.083
**Sphericity**	0.74 (0.08)	0.76 (0.09)	.062
**Surface Area**	3.75 (2.26)	5.10 (3.06)	.001*
**Surface Area Density**	7.42 (0.99)	6.42 (1.32)	<.001*
**Volume**	0.43 (0.36)	0.76 (0.66)	<.001*
**Energy**	679498.11 (240590.86)	825260.67 (288103.42)	.001*
**Global Entropy**	5.41 (0.31)	5.60 (0.32)	<.001*
**Global Max**	1782.34 (434.75)	1975.22 (514.18)	.01*
**Global Mean**	772.96 (137.85)	853.45 (163.34)	.001*
**Global Median**	757.02 (135.13)	835.38 (167.41)	.001*
**Global Min**	103.35 (146.03)	168.83 (203.80)	.016*
**Global SD**	245.63 (56.96)	257.76 (67.71)	.215
**Global Uniformity ($\times 10^{-3}$)**	29.50 (6.55)	26.28 (6.45)	.002*
**Inter-Quartile Range**	264.57 (78.22)	303.47 (102.55)	.006*
**Kurtosis**	6.22 (6.87)	5.47 (2.85)	.424
**Mean Absolute Deviation**	179.15 (44.82)	194.07 (54.59)	.055
**Median Absolute Deviation**	129.25 (39.05)	149.00 (48.90)	.004*
**5th Percentile**	404.85 (172.04)	476.55 (201.49)	.015*
**10th Percentile**	501.51 (147.53)	558.43 (190.36)	.031*
**15th Percentile**	558.37 (135.52)	613.65 (186.69)	.027*
**20th Percentile**	598.02 (134.88)	655.67 (181.72)	.019*
**25th Percentile**	631.37 (130.94)	689.80 (176.27)	.014*
**30th Percentile**	660.60 (130.24)	719.38 (173.12)	.013*
**35th Percentile**	685.85 (130.65)	750.12 (171.04)	.006*
**40th Percentile**	710.28 (131.56)	777.81 (168.02)	.004*
**45th Percentile**	733.76 (133.44)	805.23 (166.63)	.002*
**50th Percentile**	757.02 (135.13)	835.38 (167.41)	.001*

**55th Percentile**	780.66 (136.49)	861.87 (167.37)	.001*
**60th Percentile**	804.74 (140.02)	890.38 (167.07)	<.001*
**65th Percentile**	832.61 (143.11)	921.49 (166.73)	<.001*
**70th Percentile**	862.02 (146.49)	956.86 (169.28)	<.001*
**75th Percentile**	895.93 (153.36)	993.28 (171.21)	<.001*
**80th Percentile**	936.38 (159.49)	1036.46 (173.98)	<.001*
**85th Percentile**	989.67 (169.83)	1092.84 (175.59)	<.001*
**90th Percentile**	1062.09 (181.08)	1167.95 (178.68)	<.001*
**95th Percentile**	1189.53 (200.68)	1299.76 (197.81)	.001*
**0.025 Quantile**	308.01 (184.92)	396.75 (209.81)	.005*
**0.25 Quantile**	631.37 (130.94)	689.80 (176.27)	.014*
**0.5 Quantile**	757.02 (135.13)	835.38 (167.41)	.001*
**0.75 Quantile**	895.93 (153.36)	993.28 (171.21)	<.001*
**0.975 Quantile**	1326.05 (224.73)	1431.10 (215.40)	.004*
**Range**	1678.99 (435.02)	1806.40 (575.41)	.104
**Root Mean Square**	812.87 (137.46)	895.11 (156.39)	<.001*
**Skewness**	0.62 (0.92)	0.70 (0.62)	.541
**Variance**	63551.22 (30978.77)	70943.06 (38373.16)	.172
**5th Percentile Area**	404.15 (158.77)	473.67 (192.15)	.013*
**10th Percentile Area**	491.27 (147.75)	558.14 (177.97)	.01*
**15th Percentile Area**	548.96 (136.60)	615.63 (169.14)	.006*
**20th Percentile Area**	588.56 (134.48)	647.65 (181.20)	.016*
**25th Percentile Area**	622.57 (131.24)	680.63 (176.83)	.015*
**30th Percentile Area**	651.60 (130.51)	710.93 (173.58)	.012*
**35th Percentile Area**	677.15 (131.30)	741.30 (170.37)	.007*
**40th Percentile Area**	701.58 (131.49)	769.15 (167.82)	.004*
**45th Percentile Area**	724.67 (132.73)	797.04 (166.89)	.002*
**50th Percentile Area**	748.35 (135.30)	826.20 (166.35)	.001*
**55th Percentile Area**	772.50 (136.54)	853.73 (167.07)	.001*
**60th Percentile Area**	796.29 (139.92)	882.19 (167.10)	<.001*
**65th Percentile Area**	824.25 (143.06)	913.01 (166.41)	<.001*
**70th Percentile Area**	853.66 (147.34)	947.02 (167.79)	<.001*
**75th Percentile Area**	887.79 (153.28)	984.29 (171.02)	<.001*
**80th Percentile Area**	927.83 (159.46)	1027.50 (174.14)	<.001*
**85th Percentile Area**	980.43 (169.19)	1083.89 (176.14)	<.001*
**90th Percentile Area**	1051.44 (181.19)	1158.16 (177.94)	<.001*
**95th Percentile Area**	1175.54 (198.14)	1286.80 (196.50)	.001*
**Auto-Correlation (d1)**	1449.13 (510.09)	1729.69 (635.05)	.002*
**Auto-Correlation (d4)**	1447.35 (530.20)	1758.11 (643.36)	.001*
**Auto-Correlation (d7)**	1438.97 (612.98)	1767.98 (697.42)	.002*
**Cluster Prominence (d1)**	505770.73 (539381.58)	611964.36 (639431.43)	.25
**Cluster Prominence (d4)**	352494.47 (447350.31)	408213.58 (429690.38)	.433
**Cluster Prominence (d7)**	358260.02 (486411.26)	408486.31 (376054.61)	.49

**Cluster Shade (d1)**	2175.08 (4525.58)	2658.64 (4668.99)	.511
**Cluster Shade (d4)**	1788.65 (3991.08)	1968.15 (3217.24)	.766
**Cluster Shade (d7)**	2096.99 (4335.26)	2240.15 (3307.72)	.825
**Cluster Tendency (d1)**	300.21 (165.09)	347.26 (211.97)	.108
**Cluster Tendency (d4)**	267.48 (138.38)	303.33 (169.77)	.136
**Cluster Tendency (d7)**	288.92 (169.40)	303.44 (163.07)	.589
**Contrast (d1)**	135.23 (62.61)	149.01 (81.35)	.217
**Contrast (d4)**	261.20 (132.88)	257.29 (129.88)	.854
**Contrast (d7)**	423.97 (338.10)	333.92 (202.14)	.063
**Correlation (d1)**	0.37 (0.14)	0.39 (0.11)	.277
**Correlation (d4)**	0.06 (0.15)	0.11 (0.14)	.06
**Correlation (d7)**	0.01 (0.25)	0.01 (0.21)	.85
**Difference Entropy (d1)**	4.45 (0.31)	4.51 (0.36)	.233
**Difference Entropy (d4)**	4.78 (0.31)	4.85 (0.35)	.143
**Difference Entropy (d7)**	4.22 (0.88)	4.68 (0.62)	$.001^{*}$
**Dissimilarity (d1)**	8.26 (1.95)	8.63 (2.37)	.273
**Dissimilarity (d4)**	12.08 (3.24)	12.06 (3.22)	.966
**Dissimilarity (d7)**	15.40 (6.97)	13.80 (4.72)	.116
**Energy (d1) ($\times 10^{-3}$)**	2.99 (1.32)	2.63 (6.45)	$.004^{*}$
**Energy (d4) ($\times 10^{-3}$)**	6.73 (5.02)	4.19 (4.30)	$.001^{*}$
**Energy (d7)**	0.05 (0.11)	0.02 (0.04)	.051
**Entropy (d1)**	8.95 (0.59)	9.36 (0.58)	$<.001^{*}$
**Entropy (d4)**	7.67 (0.99)	8.44 (0.96)	$<.001^{*}$
**Entropy (d7)**	5.67 (1.78)	6.97 (1.64)	$<.001^{*}$
**Homogeneity (d1) ($\times 10^{-3}$)**	224.96 (35.29)	222.80 (35.86)	.705
**Homogeneity (d4) ($\times 10^{-3}$)**	167.20 (34.79)	165.51 (32.78)	.757
**Homogeneity (d7)**	0.14 (0.05)	0.15 (0.04)	.696
**Homogeneity 2 (d1) ($\times 10^{-3}$)**	140.22 (32.31)	139.01 (31.56)	.813
**Homogeneity 2 (d4) ($\times 10^{-3}$)**	90.49 (28.91)	88.98 (26.33)	.736
**Homogeneity 2 (d7)**	0.07 (0.04)	0.08 (0.03)	.715
**InfoMeasureCorr 1 (d1)**	-0.19 (0.06)	-0.17 (0.07)	.099
**InfoMeasureCorr 1 (d4)**	-0.42 (0.15)	-0.34 (0.16)	$<.001^{*}$
**InfoMeasureCorr 1 (d7)**	-0.68 (0.19)	-0.55 (0.21)	$<.001^{*}$
**InfoMeasureCorr 2 (d1)**	0.90 (0.06)	0.88 (0.08)	.079
**InfoMeasureCorr 2 (d4)**	0.98 (0.05)	0.95 (0.09)	$.003^{*}$
**InfoMeasureCorr 2 (d7)**	0.99 (0.09)	0.98 (0.05)	.74
**InvDifMN (d1) ($\times 10^{-3}$)**	987.51 (5.48)	986.23 (7.09)	.211
**InvDifMN (d4) ($\times 10^{-3}$)**	976.39 (11.14)	976.70 (11.02)	.861
**InvDifMN (d7)**	0.95 (0.09)	0.95 (0.13)	.927
**InvDifN (d1)**	0.93 (0.01)	0.93 (0.02)	.297
**InvDifN (d4) ($\times 10^{-3}$)**	899.51 (23.14)	899.48 (23.01)	.994
**InvDifN (d7)**	0.87 (0.09)	0.87 (0.12)	.938
**Inverse Variance (d1) ($\times 10^{-3}$)**	143.41 (32.79)	139.20 (33.19)	.427

**Inverse Variance (d4) (×10^{-3})**	94.09 (30.05)	91.65 (29.93)	.613
**Inverse Variance (d7)**	0.08 (0.05)	0.08 (0.04)	.783
**Max Probability (d1) (×10^{-3})**	11.50 (5.57)	11.13 (9.66)	.749
**Max Probability (d4)**	0.02 (0.01)	0.01 (0.01)	<.001*
**Max Probability (d7)**	0.06 (0.10)	0.02 (0.04)	.03*
**Sum Average (d1)**	73.96 (12.73)	80.52 (15.54)	.003*
**Sum Average (d4)**	74.91 (13.16)	82.16 (15.52)	.001*
**Sum Average (d7)**	74.98 (16.45)	82.07 (19.33)	.012*
**Sum Entropy (d1)**	5.89 (0.36)	6.02 (0.38)	.027*
**Sum Entropy (d4)**	5.58 (0.38)	5.79 (0.40)	.001*
**Sum Entropy (d7)**	4.68 (1.09)	5.30 (0.85)	<.001*
**Sum Variance (d1)**	5093.68 (1895.45)	6135.30 (2356.67)	.002*
**Sum Variance (d4)**	5243.58 (2006.88)	6372.34 (2397.90)	.001*
**Sum Variance (d7)**	5545.49 (2306.64)	6677.76 (2539.47)	.004*
**Variance (d1)**	300.21 (165.09)	347.26 (211.97)	.108
**Variance (d4)**	267.48 (138.38)	303.33 (169.77)	.136
**Variance (d7)**	288.92 (169.40)	303.44 (163.07)	.589
**Busyness (×10^{-3})**	0.99 (3.64)	0.16 (0.22)	.295
**Coarseness (×10^{-5})**	6.31 (8.17)	11.62 (8.27)	.373
**Complexity**	213192.64 (266248.09)	506720.89 (449132.79)	<.001*
**Contrast 1**	20071.28 (39605.39)	14832.72 (31136.79)	.388
**Texture Strength**	440.77 (392.87)	548.91 (439.88)	.11
**GLN (×10^{-3})**	0.21 (0.13)	0.13 (0.10)	<.001*
**HGRE**	1605.57 (548.39)	1887.22 (629.95)	.003*
**LRE**	1.24 (0.11)	1.26 (0.11)	.303
**LRHGE**	1923.14 (602.32)	2310.22 (825.89)	.001*
**LRLGE**	0.02 (0.03)	0.02 (0.07)	.608
**LGRE (×10^{-3})**	9.40 (11.59)	7.70 (14.13)	.395
**RLN (×10^{-3})**	5.12 (2.71)	3.62 (2.30)	<.001*
**RP**	0.93 (0.02)	0.92 (0.03)	.344
**SRE (×10^{-3})**	950.79 (17.13)	948.90 (15.54)	.489
**SRHGE**	1539.15 (538.87)	1803.32 (603.77)	.004*
**SRLGE (×10^{-3})**	8.14 (9.20)	5.94 (8.99)	.136

Note. — ADC, apparent diffusion coefficient; LN, lymph node; Other abbreviations, summarized in Table 1. a. All values are expressed as mean (standard deviation). b. The significance threshold for difference was set at a *P* value less than .05, according to independent *t*-test. * means statistically significant.
